# Editorial: Biomarkers of Immune-Checkpoint-Inhibitor Immunotherapies in Hepatocellular Carcinomas

**DOI:** 10.3389/fonc.2022.860964

**Published:** 2022-03-10

**Authors:** Zhanjun Guo, Heng-Hong Li, Yih-Horng Shiao

**Affiliations:** ^1^ Department of Immunology and Rheumatology, The Fourth Hospital of Hebei Medical University, Shijiazhuang, China; ^2^ Department of Oncology, Lombardi Comprehensive Cancer Center, Georgetown University, Washington, DC, United States; ^3^ United Department of Commerce, States Patent and Trademark Office, Alexandria, VA, United States

**Keywords:** Hepatocellular carcinomas, biomarkers, immune-checkpoint inhibitors, PD-1, PD-L1, immunotherapies, bioinformatics

Hepatocellular carcinomas (HCCs), made up of 75-85% of primary liver cancers, remain the leading cause of new cancer cases (>0.7 million) and cancer deaths (>0.6 million) globally according to the data collected up to 2020 before the coronavirus disease 2019 (COVID-19) pandemic (1). The etiology of HCCs has been gradually shifted from hepatitis viruses to metabolic diseases, caused by heavy alcohol intake, fatty liver, obesity, and diabetes, in part due to a global campaign in hepatitis B vaccination and effective anti-hepatitis C medication but also ineffective control of lifestyle and dietary changes that exacerbate the metabolic imbalance. The impact of COVID-19 for the next cancer report is not clear but the pandemic-related delay in cancer screening and cancer care is likely to increase diagnoses of advanced stages of HCCs. HCCs are heterogeneous in nature, which contributes to the high recurrence rate after surgical resection, the first-line cancer treatment for HCCs (2). For unresectable HCCs, targeted immunotherapies become the next-line treatment option; however, improvement of overall survival is limited and the median overall survival remains near to or less than 1 year after standard monotherapy (3). Combination therapies targeting two immune checkpoint regulators (PD-1 and CTLA-4) or PD-1 concurrently with the angiogenic pathway prolong the overall survival time to nearly twice that of patients receiving monotherapy (3; also reviewed by He et al. below). The overall survival can be further improved by stratifying the patients according to biomarkers. Widely available public genomic and proteomic databases (4, 5) facilitate the discovery of new biomarkers for assisting in treatment decisions and targeted therapy. The articles published in this Research Topic utilized these databases and their contributions to the field are discussed below.

Cai et al. constructed a mathematical model from 5 of 64 intergenic long non-coding RNAs (lncRNAs), termed an immune-related enhancer RNA signature (IReRS), which not only associated with immune pathway activation and immune-cell infiltration, but also predicted poor survival. The initial 64 lncRNAs were identified by correlating immune-related genes of the Molecular Signatures Database with expression and clinical data of HCCs from The Cancer Genome Atlas (TCGA). The predictive value of the IReRS was first obtained in a randomized training set of 224 patients and subsequently validated in a test set of 142 patients or a combination of the two sets, strengthening its predictive power.

Wei et al. demonstrated for the first time upregulation of FAM57A in HCCs and its associations with tumor stage/grade, immune-checkpoint activation, immune-cell infiltration, and poor survival. The upregulation was first discovered by comparing the transcriptome of over 700 HCC tumor samples with that of over 260 non-tumor samples in Gene Expression Omnibus (GEO), International Cancer Genome Consortium (ICGC), TCGA, and Cancer Cell Line Encyclopedia databases, and was further confirmed by immunohistochemical analyses of 30 pairs of HCCs and adjacent non-tumor tissues. *In vitro* knockdown of FAM57A reduced the HCC cell number by growth inhibition and apoptotic cell death, identifying a potential therapeutic target.

Zhou et al. indicated that six immune-related lncRNAs including *MSC-AS1, AC145207.5, SNHG3, AL365203.2, AL031985.3*, and *NRAV* were upregulated at advanced grade, tumor-stage, and T-stage. They found that the increased levels of lncRNAs were significantly associated with lower overall survival, and a signature based on the risk score of the six lncRNAs was an independent prognostic factor in patients suffering from HCC. The signature of immune-related lncRNAs was also associated with immune-cell infiltration in HCC. The research showed that patients with a high signature score of immune-related lncRNAs also had a high level of immune checkpoint genes and these patients presented worse survival rates than those with a low signature score.

Qi et al. used some tumor mutation burden (TMB)-associated lncRNAs to establish an eight-lncRNA prognostic signature, which was subsequently validated as a independent prognostic factors for HCC. Patients with a high score in the eight-lncRNA prognostic signature tended to possess increased immune infiltration and TMB. In addition, the finding was further confirmed in a competitive endogenous RNA (ceRNA) network named LINC00638/miR-4732-3p/ULBP1, which was newly constructed by the investigators.

Zhu et al. identified *LINC00485* as a potential diagnostic and prognostic biomarker for HCC. *LINC00485* was selected by screening the TCGA database, and the increase of *LINC00485* level was found in serum samples of HCC patients. Combination of *LINC00485* with alpha fetoprotein (AFP) improved the detection rate of HCC. To explore the possible mechanisms of *LINC00485* to HCC progression, a lncRNA-miRNA-mRNA regulatory network was proposed based on analysis using lncRNA and miRNA target prediction tools.

In the review article entitled “*Biomarkers and Future Perspectives for Hepatocellular Carcinoma Immunotherapy*”, the authors briefly summarized the clinical trials in the treatment of HCC using immune checkpoint inhibitors (ICI), reviewed potential predictive biomarkers for response to ICI-based treatment, and discussed potential biomarkers for ICI treatment-related adverse events.

## Author Contributions

ZG, H-HL, and Y-HS drafted and revised the manuscript. All authors contributed to the article and approved the submitted version.

## Conflict of Interest

The authors declare that the research was conducted in the absence of any commercial or financial relationships that could be construed as a potential conflict of interest. The views expressed do not necessarily represent those of the US Federal agency.

## Publisher’s Note

All claims expressed in this article are solely those of the authors and do not necessarily represent those of their affiliated organizations, or those of the publisher, the editors and the reviewers. Any product that may be evaluated in this article, or claim that may be made by its manufacturer, is not guaranteed or endorsed by the publisher.
